# Estrogen receptor beta expression in triple negative breast cancers is not associated with recurrence or survival

**DOI:** 10.1186/s12885-023-10795-5

**Published:** 2023-05-19

**Authors:** Elena A. Takano, Melissa M. Younes, Katie Meehan, Lisa Spalding, Max Yan, Prue Allan, Stephen B. Fox, Andy Redfern, David Clouston, Graham G. Giles, Elizabeth L. Christie, Robin L. Anderson, Magnus Zethoven, Kelly-Anne Phillips, Kylie Gorringe, Kara L. Britt

**Affiliations:** 1 Pathology, Peter MacCallum Cancer Centre, Melbourne, VIC 3000 Australia; 2 Breast Cancer Risk and Prevention Laboratory, Peter MacCallum Cancer Centre, Research Division 305 Grattan St, Melbourne, VIC 3000 Australia; 3 Department of Otorhinolaryngology, Head and Neck Surgery, Faculty of Medicine, The Chinese University of Hong Kong, Shatin, Hong Kong; 4 The University of Western Australia (M504), 35 Stirling Highway, Perth, 6009 Australia; 5 South Eastern Area Laboratory Services, Randwick, NSW Australia; 6 School of Cancer Medicine, La Trobe University, Bundoora, VIC 3086 Australia; 7 TissuPath, 32 Ricketts Rd, Mount Waverley, VIC 3149 Australia; 8 7a Cancer Epidemiology Division, Cancer Council Victoria, Melbourne, VIC 3004 Australia; 9 Centre for Epidemiology and Biostatistics, School of Population and Global Health, University of Melbourne, Parkville, VIC 3012 Australia; 10 Precision Medicine, School of Clinical Sciences at Monash Health, Monash University, Clayton, VIC 3168 Australia; 11 Peter MacCallum Cancer Centre Melbourne, Victoria, 3000 Australia; 12 Olivia Newton-John Cancer Research Institute, Heidelberg, VIC 3084 Australia; 13 The Sir Peter MacCallum Department of Oncology, University of Melbourne, Parkville, VIC Australia; 14 Peter Mac, Bioinformatics Core Facility, Peter MacCallum Cancer Centre, 305 Grattan St, Melbourne, VIC Australia; 15 Department of Medical Oncology, Peter MacCallum Cancer Centre, Melbourne, VIC Australia; 16 Centre for Epidemiology and Biostatistics, School of Population and Global Health, The University of Melbourne, Parkville, VIC Australia; 17 Precision Cancer Medicine Laboratory, Peter MacCallum Cancer Centre, 305 Grattan St, Melbourne, VIC Australia

**Keywords:** Estrogen receptor beta, Triple negative breast cancer, Tamoxifen, Prognosis, Outcome, Sensitivity

## Abstract

**Background:**

Triple negative BCa (TNBC) is defined by a lack of expression of estrogen (ERα), progesterone (PgR) receptors and human epidermal growth factor receptor 2 (HER2) as assessed by protein expression and/or gene amplification. It makes up ~ 15% of all BCa and often has a poor prognosis. TNBC is not treated with endocrine therapies as ERα and PR negative tumors in general do not show benefit. However, a small fraction of the true TNBC tumors do show tamoxifen sensitivity, with those expressing the most common isoform of ERβ1 having the most benefit. Recently, the antibodies commonly used to assess ERβ1 in TNBC have been found to lack specificity, which calls into question available data regarding the proportion of TNBC that express ERβ1 and any relationship to clinical outcome.

**Methods:**

To confirm the true frequency of ERβ1 in TNBC we performed robust ERβ1 immunohistochemistry using the specific antibody CWK-F12 ERβ1 on 156 primary TNBC cancers from patients with a median of 78 months (range 0.2–155 months) follow up.

**Results:**

We found that high expression of ERβ1 was not associated with increased recurrence or survival when assessed as percentage of ERβ1 positive tumor cells or as Allred > 5. In contrast, the non-specific PPG5-10 antibody did show an association with recurrence and survival.

**Conclusions:**

Our data indicate that ERβ1 expression in TNBC tumours does not associate with prognosis.

**Supplementary Information:**

The online version contains supplementary material available at 10.1186/s12885-023-10795-5.

## Background

Estrogen actions in tissues are mediated by two structurally related but genetically distinct receptors, estrogen receptor (ER) α and ERβ [1, 2]. In the normal breast, ERα is expressed in a modest subset of luminal epithelial cells where it mediates proliferation and breast growth. ERα expression is common in breast cancer (BCa), with 75% of tumors being ERα positive. It is an important biomarker for response to anti-estrogen therapy and is widely used in diagnosis and treatment planning. On the other hand, ERβ is more abundant than ERα in normal mammary tissue [3] and expression is often diminished or lost in BCa [4–6]. ERβ exists as five isoforms, ERβ1-5, but isoforms 2–5 are C-terminally truncated and cannot bind ligands [7, 8] leaving only ERβ1 as the functional receptor for estrogen ligand action.

Triple negative breast cancer (TNBC) is defined by a lack of expression of ERα, PgR and HER2. These tumors make up 10–15% of all BCa and characteristically recur early with the peak risk of recurrence and the majority of deaths occurring within the first three and five years after the initial treatment, respectively [9, 10]. They are associated with an inferior prognosis despite their greater sensitivity to cytotoxic chemotherapies in the neo-advujant, adjuvant and later in the metastatic settings. Thus, additional targeted therapies for TNBC are needed.

Tamoxifen is a selective ER modulator (SERM) that competitively binds to ERα and blocks estrogen binding. It is prescribed for the treatment of ERα + BCa due to its ability to inhibit estrogen-stimulated proliferation in cancer cells. There is a small benefit for the rare ERα negative, PR positive cancers and guidelines recommend that these patients be given endocrine therapy. Patients with ERα negative PR negative tumors in general do not benefit from tamoxifen therapy, although a modest proportion (5–10%) show sensitivity to tamoxifen [11, 12].

Tamoxifen action on signaling targets other than ERα has been proposed as a mechanism to explain sensitivity in ERα negative tumors. Three studies have shown that ERβ1 expression acts as a marker for favorable prognosis in tamoxifen-treated ERα-negative [13–15] and TNBC patients [14] indicating that ERβ1 is clinically relevant. However, a lack of placebo treated patients for comparison in two studies [13, 14] prevented robust establishment of whether ERβ1 acts as a general prognostic marker or as a predictor of tamoxifen sensitivity.

Numerous studies have assessed the frequency of ERβ1 expression in TNBC. The spectrum of ERβ1 positivity in these studies ranges from 35 to 75% [13, 14, 16–19]. However, two recent publications have questioned the specificity of many previously employed ERβ antibodies, including the PPG5/10 ERβ1 antibody used in most studies [20, 21]. In both immunohistochemistry (IHC) and western blotting, the PPG5/10 ERβ1 antibody, which targets the carboxterminal end of ERβ1, demonstrated low specificity, showing positivity in ERβ1-negative control lines [20, 21]. This calls into question the validity of data from existing studies of TNBC, which have largely used the PPG5/10 antibody.

Nelson et al. used antibody-dependent (IHC and western blotting) as well as antibody-independent (RT-qPCR) analysis to confirm the specificities of multiple antibodies and validated MC10 (targets the N-terminus) and CWK-F12 (targets the ligand binding domain) antibodies as being specific for ERβ1 [21]. Rapid immunoprecipitation mass spectrometry of endogenous protein (RIME) analysis identifies the specificity and peptide coverage of antibodies, including ERβ1, without the need of another antibody dependent technique such as western blotting, where one must rely on the migration mobility of a band. CWK-F12 also performed very well in the RIME analysis and demonstrated differential IHC nuclear staining of ERβ1 between MDA-MB-231 cells with inducible exogenous ERβ1 expression and control cells [21].

In view of this, we sought to determine the true percentage of TNBC that express ERβ1 and any relationship with clinical outcome using CWK-F12 antibody [20–22].

## Methods

### Tissue microarrays (TMA) of TNBC patients

To analyse the frequency of ERß1 in TNBC samples, protein levels were determined using two independent TNBC TMAs. Cases that were included were all TNBC cases (stage 1–3) that were coming through the clinic and were pathologically ER negative, PR negative and HER2 negative. All cases were negative for ER-alpha (0% staining) except for 2 cases which were < 1% weak staining. Thus, all are considered negative under the historical guidelines (2000–2010; < 10% staining) and revised 2010 ASCO/CAP guidelines (< 1% staining). The first TMA was obtained from the Peter MacCallum Cancer Centre (PMCC) and contained 1 mm cores from 70 human primary TNBC tumors. Tissue samples were obtained from the PMCC, Royal Melbourne Hospital, St Vincent’s Hospital and Monash Health from women undergoing breast surgery between 2004 and 2011. The median age at diagnosis was 60 years and patients had a median follow-up of 72 months (range 0.2–137 months). The second TMA was obtained from Perth, Western Australia, containing 1 mm cores from 97 primary breast tumors. Tissue samples were retrieved from Sir Charles Gairdner Hospital (SCGH) from women undergoing breast surgery between 2005 and 2013. Of these, 9 cases did not have successful staining (no core after IHC, or not enough tumour in the core) and were removed from further analysis. We also removed another 2 samples as followup was too short or one core was a replicate of an exisiting core. The median age at diagnosis of the remaining 56 samples was 59 years with a median follow-up of 84 months (range 5–155 months). When both cohorts combined, the median age at diagnosis was 59 years and the median follow-up was 78 months (range 0.2–155 months). Table 1 shows the demographics and characteristics for the cohorts. Our cohorts precede the more widespread utilization of newer agents such as checkpoint immunotherapy and Sacituzumab for TNBC patients. Information on breast cancer recurrence and death came from medical records and the respective state cancer registries for Victoria and WA. For the combined cohort (*n* = 156), 36% (55/156) had a recurrence. The percentage of patients that died was 35% (54/156) and this was mainly death due to BCa (42/54) rather than other causes (11/54) or unknown (1/54).

**Table 1:** Patient demographics and characteristics according to ERβ expression

	**ERb High (> = 40%)** N (%*)	**ERb Low (< 40%)** N (%*)	***p*** **-value** **Chi sq**
**Total Patients**			
Total	81	75	< 0.00001
PMCC	51 (63)	19 (25)	
WA	30 (37)	56 (75)	
**Age**			
Median age	62	56	0.0073
< = 50	14 (17)	30 (40)	
> 50–70	44 (54)	28 (37)	
> 70	23 (28)	17 (23)	
**Grade**			
1	0 (0)	0 (0)	0.659
2	4 (5)	5 (7)	
3	77 (95)	70 (93)	
**Tumour Size**			
Median size	25	25	0.613
T1 (1–19)	30 (37)	24 (32)	
T2 (20–49)	45 (56)	46 (61)	
T3 (50–99)	5 (6)	3 (4)	
T4 (100 +)	0 (0)	0 (0)	
unknown	1 (1)	2 (3)	
**LN status**			
NO	27 (33)	27 (36)	0.947
N1 +	44 (64)	43 (57)	
unknown	10 (12)	5 (7)	
**Stage**			
1A	22 (27)	19 (25)	0.931
IIA	30 (37)	28 (37)	
IIB	12 (15)	14 (19)	
III/IV	13 (16)	11 (15)	
Unknown	4 (5)	3 (4)	
**Recurrence**			
No	47 (58)	54 (72)	0.089
Yes	34 (42)	21 (28)	
**Mortality**			
Yes (Death)	29 (36)	25 (33)	0.834
No (Alive)	52 (64)	50 (67)	

% calculated from patients with known value. * *P*<0.05

### Ethics approval for human samples

The PMCC (03/90, 00/81) and SCGH cohorts received ethics approval from their local ethical review boards to collect and share samples and clinical data. Patients had either given broad written consent to future research with their samples and data, or waivers of consent were in place. The research assessing estrogen receptor beta was approved by the Peter MacCallum Human ethics committee (10_16 and 21_76). The study was conducted in accordance with the Australian National Health and Medical Research statement on ethical conduct in human research. The study was performed in accordance with the Declaration of Helsinki.

### Immunohistochemical staining and scoring

The level of ERß1 was analysed using the CWK-F12 ERß1 antibody (*Developmental Studies Hybridoma Bank,* DSHB) using IHC. As discussed, validation using RIME showed this antibody to be ERß1 specific [21] in addition to which we have previously validated its specificity using IHC [22]. ERß1 IHC was performed on an automated IHC slide staining system, Ventana BenchMark Ultra (Roche Diagnostics, USA). Briefly, 3 µm thick FFPE sections mounted on coated slides (Series 2 Adhesive, Trajan Scientific Australia) were de-waxed and antigen retrieved in ULTRA Cell Conditioning Solution 2 (CC2, Roche Diagnostics) for 40 min at 97 °C. Following incubation in the OptiView Peroxidase Inhibitor (Roche Diagnostics, USA) for 5 min at room temperature, the sections were incubated in the ERß1 antibody, CWK-F12 (DSHB Hybridoma) at 0.14 µg/ml (1:320) for cell pellets or at 1.1 µg/ml (1:40) for tissue sections for 60 min at room temperature. On-board detection system, OptiView Universal DAB Detection Kit (Roche Diagnostics, USA), was used in a visualization step in accordance with the manufacturer’s instructions.

For exploratory analysis allowing comparison to previous work, we also assessed expression of ERß1 using the PPG5/10 antibody. The DAKO EnVision FLEX high pH kit was used with the ERß1 PPG5/10 (GeneTex) antibody diluted at 1:15. Scoring was performed by a breast pathologist (PA) and independently confirmed by a second scorer (KB). Both scorers were blinded to the clinical characteristics of the tumor samples. Cores were scored for the percentage of ERß1 positive tumor cells, as well as the intensity of staining to generate an Allred score that incorporates both aspects. Intensity was scored as negative = 0, weak = 1, moderate = 2 or strong = 3, and the percentage of positively stained tumor cells was classified as: 0% = 0; < 1% = 1, 1–10% = 2, 11–33% = 2, 34–66% = 4, 67–100% = 5. Scores were added to form a maximum score of 8. ERß1 positive was defined as those tumors with 40% or more ERβ1 + cells of any intensity. To determine the most appropriate cut-off of expression for our analysis with clinicopathological features, we assessed the distribution data from Allred scores and ERβ1 expression percentages (vs frequency) as recommended from past studies [23]. A mixture model of two Gaussian distributions is fitted to the histogram of the expression using the *flexmix* function in R. The optimal cutoff is determined as the value where the probability density functions of the mixing distribution coincide.

### Statistical analysis

Survival analysis was performed in RStudio (v1.1.453, running R v4.0.3). Descriptive statistics were used to assess the proportion of TNBCs that were ERß1 positive and the association of ERß1 with BCa recurrence and survival. BCa recurrence was defined as locoregional and distant recurrence). Overall and breast cancer-specific survivals were assessed. Cox proportional hazards model was used to determine the association of variables with survival (*survival::coxph*), and univariate models were visualised in a Kaplan–Meier plot (unadjusted, *survminer::ggsurvplot*). Multivariate analyses included age, tumor grade and tumor size as continuous variables, axillary lymph node status chemotherapy and ERß1 status as categorical variables and cohort as a stratifying variable. Lymph node was assessed categorically as we did not have continuous data for all patients. *survival::cox.zph* was used to test the assumptions of the multivariate Cox proportional hazards test, which were visualised by *ggcoxzph*. Akaikie Information Criterion (AIC) was also used for step-wise model selection using *MASS:stepAIC*.

### Bioinformatic analysis

#### ERß expression in TNBC subtypes

Lehmann and colleagues compiled 587 TNBC gene expression profiles from 21 studies (training set = 386 and validation set = 201. They used k-means and consensus clustering of the tumor profiles to reveal that TNBC is composed of six stable subtypes. These were Basal-like 1 (BL1), basal-like (BL2), immunomodulatory (IM), mesenchymal (M), mesenchymal stem–like (MSL), and luminal androgen receptor (LAR) subtype [24]. They later refined their classification to 4 consistent classes (TNBCtype-4)-BL1, BL2, M, and LAR [25]. We accessed the same GSE files (apart from GSE-28821, GSE-28796, GSE-22513 and GSE-18864 which were not detailed in their supplemental data) to assess the relationship of the different TNBC subtypes with ERß1 (gene symbol ESR2). Raw data (Affymetrix CEL files) were downloaded from public data repositories (GSE7390, GSE2603, MDA133, GSE3494_hgu133a, GSE2990, GSE2034, GSE11121, GSE1561, GSE7904, GSE1456_hgu133a, GSE5847, GSE20194, GSE19615, GSE5327, GSE16446, GSE12276) and files read into R and normalized using robust multi-array normalisation with the affy R package (version 1.64.0) [24] and then log-normalized. For each array dataset, probes were matched to gene symbols using the AnnotationDbi R package (version 1.48.0) and expression values were collapsed to gene-level by taking the probe for each gene with the highest interquartile range of log-expression. All arrays were then quantile normalised together using the normalize.quantiles function from the preprocessCore R package (version 1.48.0) and batch effects were removed using the removeBatchEffect function from the limma R package (version 3.42.2) [26]. TNBC samples were then identified based on the 2-component Gaussian mixture distribution model of Lehmann and colleagues [25]. *P* values and confidence intervals for the differences of the means of each gene for each pairwise comparison between the TNBC subtypes were calculated using Tukey’s range test.

#### Expression of ERß and downstream targets TNBC

The TCGA Breast Invasive Carcinoma study data was utilised, specifically the 1084 samples that have been contributed to the PanCancer Atlas study. The clinical data was downloaded from cBioPortal on 8^th^November 2022. The RNAseq data was downloaded from the ICGC Data Portal on the 17^th^ November 2022. The RNAseq RSEM raw count data was filtered for lowly expressed genes and TMM normalized to generate log CPM data using the edgeR package. The basal subtype samples (*n *= 173) were evaluated for *ESR2* and downstream gene expression. As *ESR2* RNA expression levels varied across the cohort, each sample was classified based on their *ESR2* expression level as high (top quartile), moderate (middle two quartiles) and low (bottom quartile). Pearson correlations were performed on the high *ESR2* expressing group between *ESR2* and its downstream genes expression level.

## Results

### A high proportion of TNBC express ERβ1

**Fig. 1:**
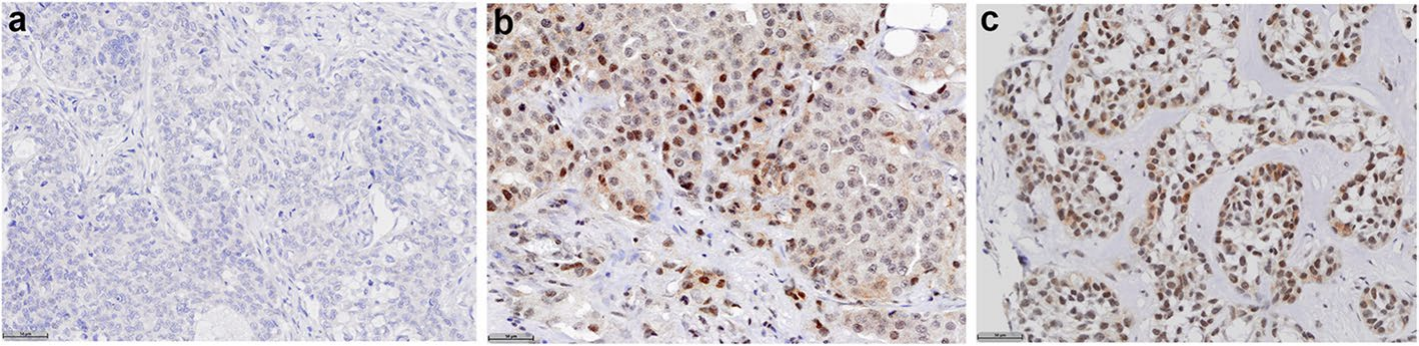
Expression of ERβ1 in TNBC TMA cores. Examples of negative (**a**) ERβ1 nuclear staining, less than (or equal to) 40% (**b**) and more than 40% (**c**). Scale bar in a represents 50 µm. The core in (**b**) had an Allred score of 3 so was within the Allred 1–4 group. The core in (**c**) had an Allred of 6 and so was within the Allred 5 + group

With much of the data on ERβ1 to date performed with non-specific antibodies, there is not clear cutoff for ERβ1 expression. We began our analysis by exploring the most appropriate scoring method. As a preliminary approach to the data, we distinguished between no ERβ1 staining as “true negative” to any ERβ1 staining as “true positive”, finding 72% of the whole cohort to be positive for any ERβ1. Examples of staining in TNBC cores are shown in Fig. 1. A stacked histogram of Allred scores or percentage of ERβ1 + cells vs frequency was computed based on the data from both cohorts. Assessing the percentage of ERβ1 + cells we found there were two clear groups, < 40% staining and > 40% staining and so these thresholds were used for analysis (Supplementary Fig. 1). In our cohort, the majority (52%) of the TNBC patients had high (> 40%) ERβ1 staining. To compare our data to previously published results, we also assessed the frequency of Allred score and found three distinguishable frequency cohorts. These cut-offs were: 0 (no ERβ1 intensity percentage), 1–5 Allred score, or > 5 Allred score (Supplementary Fig. 1).

The demographics and characteristics of the patients according to whether they had high (> 40%) or low (< 40%) ERβ1 staining are shown in Table 1.

### ERβ1 expression is not associated with recurrence in TNBC

**Fig. 2:**
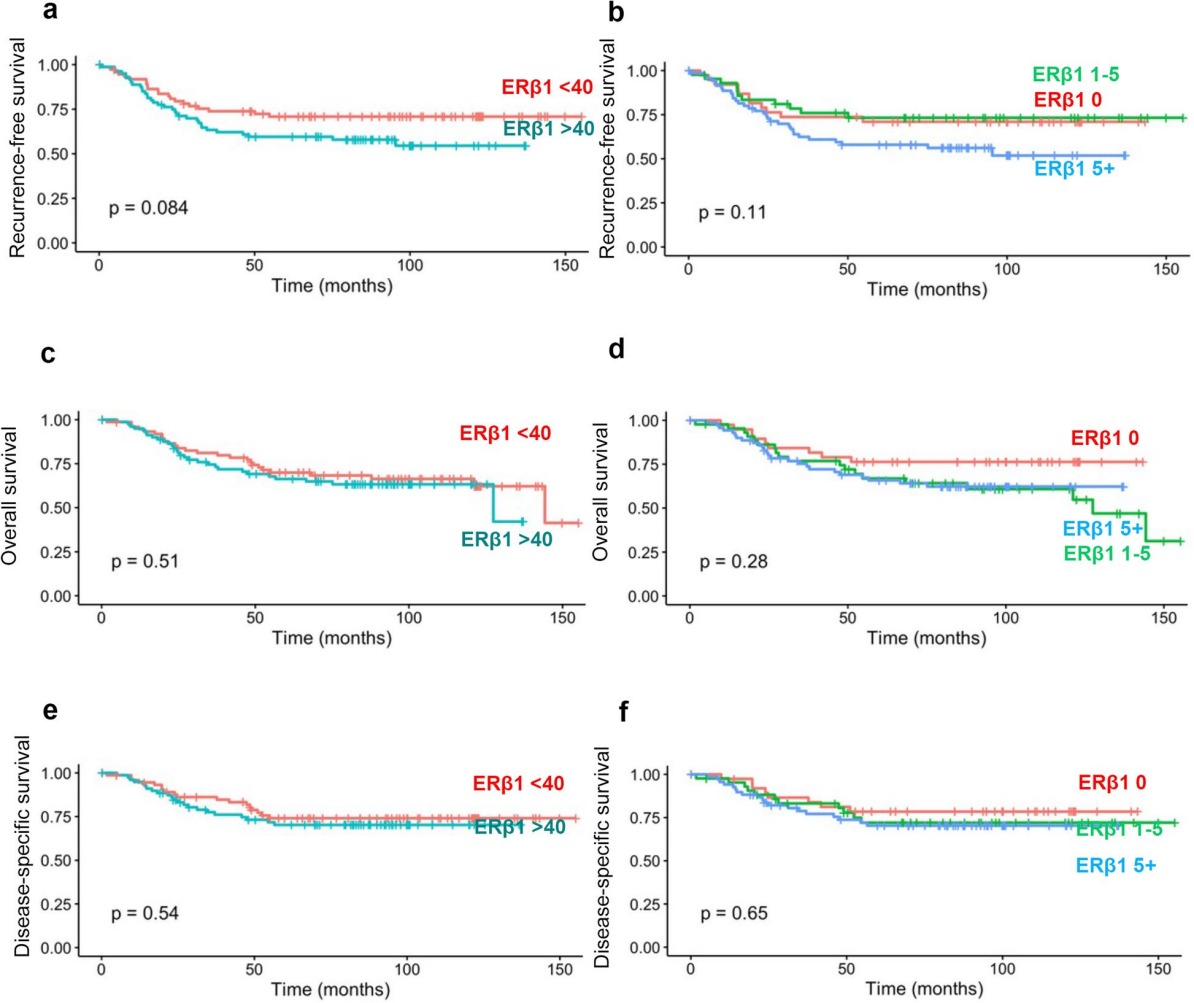
Relationship of ERβ1 expression and recurrence and survival. Kaplan Meier curves of (**a**-**b**) recurrence free survival, (**c**-**d**) overall survival and (**e**–**f**) dissease free survival according to the expression of ERβ1 when divided into (**a**, **c** and **e**) less than (or equal to) 40% or more than 40%. (**b**, **d** and **f**) Allred score of 0, 1–5 or > 5

ERβ1 high cases were slightly more likely to experience a recurrence than ERβ1 low cases but this did not reach statistical significance, *p* = 0.084 (Fig. 2a). For exploratory analysis allowing comparison to previous work, we also assessed expression according to Allred scores. Higher Allred scores did not show a significant association with prognosis *p* = 0.11 (Fig. 2b). We assessed the univariate association between BC recurrence and all available clinical features (Table 2) and found statistically significant associations with age > 50 (HR 2.01 CI[1.04–4.11]) as well as age as continuous variable (1.04 [1.02–1.06]), size (1.02 [1.00–1.04]), LN status 2.97 [1.65–5.37], Stage IIA (3.16 [1.28–7.79]) and Stage III/IV (8.00 [3.10–20.62]). Additionally Surg WLE (vs. mastectomy) 0.76 [0.43–1.33] and Chemotherapy Yes (vs. no) (0.38 [0.21–0.68]) were associated with reccurence.

**Table 2:** Univariate association between clinical and pathology features and BC recurrence and survival

	**Recurrence**		**Overall survival**		**Disease specific survival**	
**Feature (reference)**	**HR [CI 2.5–97.5%]**	***p*** ** value**	**HR [CI 2.5–97.5%]**	***p*** ** value**	**HR [CI 2.5–97.5%]**	***p*** ** value**
Age^a^	1.04 [1.02–1.06]	< 0.000005*	1.05 [1.03–1.07]	< 0.0000001*	1.04 [1.02–1.06]	0.00013*
Age > 50 (vs. < 50)	2.01 [1.04–4.11]	0.037*	2.90 [1.37–6.17]	0.0056*	1.88 [0.87–4.08]	0.11
ERβ1 > 40% (vs. ERβ1 < 40%)	1.61 [0.93–2.76]	0.086	1.20 [0.70–2.05]	0.508	1.22 [0.65–2.27]	0.538
ERβ1 Allred 1–4 (vs. ERβ1 Allred 0)	0.90 [0.39–2.08]	0.83	1.81 [0.81–4.04]	0.145	1.31 [0.53–3.26]	0.560
ERβ1 Allred > 5 (vs. ERβ1 Allred 0)	1.70 [0.85–3.39]	0.13	1.76 [0.82–3.77]	0.149	1.47 [0.64–3.36]	0.359
Size^b^	1.02 [1.00–1.04]	0.016*	1.03 [1.01–1.05]	0.0003*	1.02 [0.99–1.04]	0.063
LN status positive (vs. LN status negative)	2.97 [1.65–5.37]	0.0003*	3.30 [1.79–6.08]	0.0001*	2.91 [1.47–5.73]	0.002*
Grade 3 (vs. Grades 1 and 2)	1.32 [0.41–4.22]	0.643	2.18 [0.53–9.01]	0.283	1.45 [0.35–6.03]	0.605
Stage IIA	3.16 [1.28–7.79]	0.012*	2.51 [1.05–5.98]	0.039*	4.99 [1.46–17.07]	0.010*
Stage IIB	2.45 [0.85–7.09]	0.098	2.67 [0.98–7.23]	0.054	3.74 [0.93–14.97]	0.062
Stage III/IV	8.00 [3.10–20.62]	0.000017*	6.92 [2.76–17.34]	0.000037*	9.60 [2.67–34.60]	0.00054*
Surg WLE (vs. mastectomy)	0.76 [0.43–1.33]	0.33	0.60 [0.34–1.06]	0.079	0.56 [0.28–1.12]	0.101
RT Yes (vs. no)	0.89 [0.48–1.62]	0.679	0.86 [0.46–1.60]	0.635	0.85 [0.41–1.74]	0.648
Chemotherapy Yes (vs. no)	0.38 [0.21–0.68]	0.0011*	0.33 [0.18–0.61]	0.0004	0.42 [0.20–0.86]	0.018*

Footnote**:** ^a^Continuous (years). ^b^Continuous (mm). Recurrence is defined as locoregional or distant. Overall survival defined as death from any cause. Disease specific survival defined as death from breast cancer. * *P*<0.05

In multivariate analysis of recurrence free survival, stage IIA, stage IIB and stage III/IV were significantly associated with recurrence (Tables 3 and 4). When performing model selection using AIC, for recurrence, the combined model of LN, Age and ERβ1 > 40% (versus < 40%) and size and LN:Age (interaction between lymph node positivity and patient age) (AIC 287.02) was better able to predict recurrence than LN alone (293.74) or LN and age (289.38).

**Table 3:** Multivariate analysis of clinical features and ERβ1 expression with recurrence

**Feature (reference)**	**HR [CI 95%]**	***p*** ** value**
ERβ1 > 40% (vs. ERβ1 < 40%)	1.51 [0.76–3.01]	0.283
Age^a^	1.02 [1.02–1.05]	0.114
Stage IIA	3.23 [1.16–8.94]	0.024*
Stage IIB	3.34 [1.08–10.39]	0.036*
Stage III/IV	6.40 [2.11–19.39]	0.00102*
Chemotherapy Yes (vs. no)	0.51 [0.24–1.09]	0.0816

Footnote: ^a^Continuous (years). * *P*<0.05

**Table 4:** Multivariate analysis of clinical features and ERβ1 expression (Allred) with recurrence

**Feature (reference)**	**HR [CI 95%]**	***p*** ** value**
ERβ1 Allred 1–4 (vs. ERβ1 Allred 0)	0.57 [0.20–1.58]	0.279
ERβ1 Allred 5 + (vs. ERβ1 Allred 0)	1.04 [0.42–2.58]	0.938
Age^a^	1.02 [0.99–1.05]	0.051
Stage IIA	4.37 [1.42–13.47]	0.0101*
Stage IIB	4.24 [1.25–26.21]	0.0201*
Stage III/IV	7.78 [2.31–26.21]	0.0009*
Chemotherapy Yes (vs. no)	0.46 [0.21–1.04]	0.062

Footnote: ^a^Continuous (years). * *P*<0.05

### ERβ1 expression is not associated with worse overall survival in TNBC

No association was seen between expression of ERβ1 and overall survival *p* = 0.51 (Fig. 2c). The Allred score was also not significantly associated with overall survival, *p* = 0.28 (Fig. 2d). We assessed the univariate association between death and all available clinical features (Table 2) and found statistically significant associations with age > 50 vs < 50 (2.90 [1.37–6.17]) as well as age as a continuous variable (1.05 [1.03–1.07]) tumor size (1.03 [1.01–1.05]) and LN status; positive vs. negative (3.30 [1.79–6.08]). Additionally, stage IIA(2.51 [1.05–5.98]), Stage III/IV (6.92 [2.76–17.34]) and chemotherapy Yes (vs. no) (0.33 [0.18–0.61]) were associated with overall survival. In multivariate analyses for overall survival only age and Stage III/IV were significant (Tables 5 and 6).

**Table 5:** Multivariate analysis of clinical features and ERβ1 expression with overall survival

**Feature (reference)**	**HR [CI 95%]**	***p*** ** value**
ERβ1 > 40% (vs. ERβ1 < 40%)	1.16 [0.57–2.35]	0.681
Age^a^	1.03 [1.01–1.06]	0.0108*
Stage IIA	1.65 [0.81–6.97]	0.291
Stage IIB	2.39 [1.59–5.45]	0.112
Stage III/IV	3.01 [1.06–8.75]	0.0382*
Chemotherapy yes (vs. no)	0.56 [0.25–1.24]	0.1498

Footnote: ^a^Continuous (years). * *P*<0.05

**Table 6:** Multivariate analysis of clinical features and ERβ1 expression (Allred) with overall survival

**Feature (reference)**	**HR [CI 95%]**	***p*** ** value**
ERβ1 Allred 1–4 (vs. ERβ1 Allred 0)	1.42 [0.42–3.09]	0.793
ERβ1 Allred 5 + (vs. ERβ1 Allred 0)	1.17 [0.43–3.25]	0.752
Age^a^	1.03 [1.01–1.07]	0.004*
Stage IIA	1.96 [0.73–5.28]	0.179
Stage IIB	2.57 [0.85–7.83]	0.096
Stage III/IV	3.17 [1.06–9.44]	0.0383*

Footnote: ^a^Continuous (years). * *P*<0.05

### ERβ1 expression is not associated with disease-specific survival in TNBC

With regards to disease-specific survival we found that ERβ1 low cases had similar survival to ERβ1 high cases, *p* = 0.54 Fig. 2e). When expression was divided into Allred scores, there was no association of ERβ1 score with disease specific survival *p* = 0.65 (Fig. 2f). We assessed the univariate association between death from breast cancer and all available clinical features (Table 2) and found statistically significant associations with age (as a continuous variable) (1.04 [1.02–1.06]), tumor size (1.02 [0.99–1.04]), and LN status (positive vs. negative) (2.91 [1.47–5.73]) stage IIA(4.99 [1.46–17.07]), Stage III/IV (9.6 [2.67–34.60]) and chemotherapy Yes (vs. no) (0.42 [0.20–0.86]). In multivariate analyses for disease-specific survival only age and stage 11B and stage III/IV were significant (Tables 7 and 8), with stage IIA also significant for Allred.

**Table 7:** Multivariate analysis of clinical features and ERβ1 expression with disease specific survival

**Feature (reference)**	**HR [CI 95%]**	***p*** ** value**
ERβ1 > 40% (vs. ERβ1 < 40%)	1.13 [0.51–2.51]	0.762
Age^a^	1.03 [1.00–1.06]	0.044*
Stage IIA	3.49 [0.97–12.52]	0.056
Stage IIB	4.75 [1.17–19.36]	0.029*
Stage III/IV	4.42 [1.08–18.04]	0.038*
Chemotherapy yes (va. no)	0.61 [0.25–1.52]	0.288

Footnote: ^a^Continuous (years). * *P*<0.05

**Table 8:** Multivariate analysis of clinical features and ERβ1 expression (Allred) with disease specific survival

**Feature (reference)**	**HR [CI 95%]**	***p*** ** value**
ERβ1 Allred 1–4 (vs. ERβ1 Allred 0)	1.03 [0.34–3.19]	0.953
ERβ1 Allred 5 + (vs. ERβ1 Allred 0)	1.04 [0.34–3.24]	0.939
Age^a^	1.04 [1.01–1.07]	0.0141*
Stage IIA	5.33 [1.17–24-26]	0.0304*
Stage IIB	6.64 [1.32–33.46]	0.0217*
Stage III/IV	5.76 [1.12–33.46]	0.0344*
Chemotherapy Yes (vs no)	0.70 [0.27–1.85]	0.474

Footnote**:** ^a^Continuous (years). * *P*<0.05

### ERβ1 is not associated with a particular subtype of TNBC

**Fig. 3:**
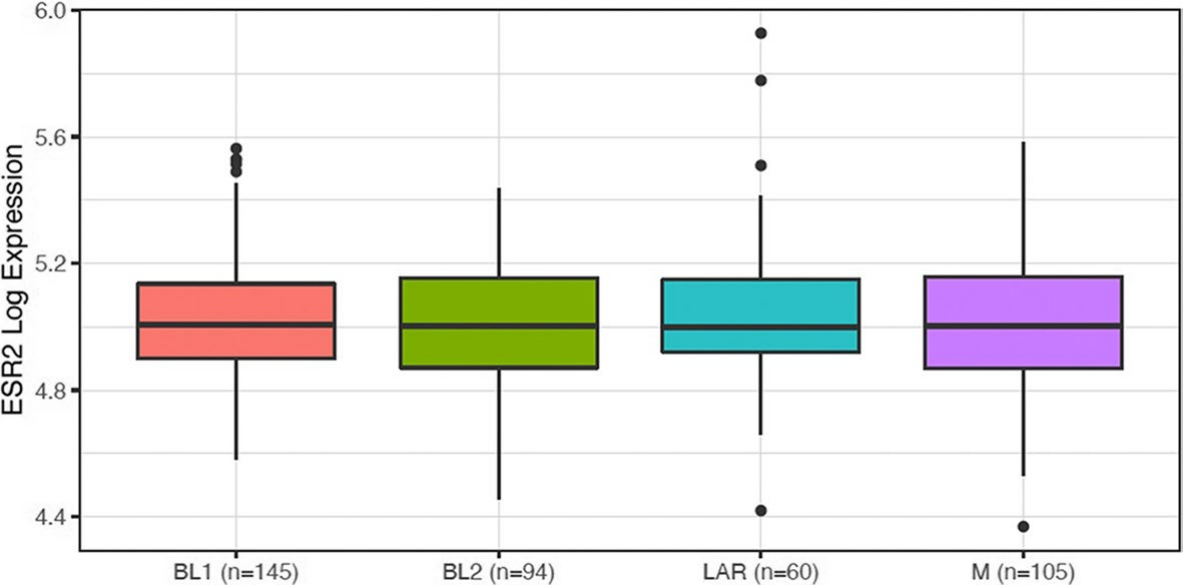
ERβ1 gene expression across different TNBC subtypes. Log expression of estrogen receptor beta (ESR2) in gene expression datasets of TNBC according to their annotated TNBC subtypes. Basal-like 1 (BL1), basal-like (BL2), immunomodulatory (IM), mesenchymal (M), mesenchymal stem–like (MSL), luminal androgen receptor (LAR) subtype and unspecified group (UNS)

Since the original studies exploring the role of ERβ1 in TNBC, much research has been completed to further characterize drivers of progression in TNBC, resulting in the identification of a number of sub-types: Basal-like 1 (BL1), basal-like (BL2), mesenchymal (M), and luminal androgen receptor (LAR) subtype [25]. There was no association of ERβ1 expression (gene symbol ESR2) with any particular subtype (Fig. 3), whilst as a positive control for the analysis, the Androgen Receptor, was significantly associated with LAR subtype as compared to the other TNBC subtypes (Supplementary Fig. 2). We also assessed expression of the ERβ gene in the previously described 6 subtypes of TNBC subtypes which also includes immunomodulatory (IM), and mesenchymal stem–like (MSL) [23] but also did not observe any association of ERβ1 with any of the TNBC (data not shown). To determine if *ESR2* mRNA was expressed in a high percentage of TNBC we assessed TCGA data and found that TNBC expressed higher levels than other BCa, as has been stated previously. This result agreed with our work showing that ESR2 expression is common (Supplementary Fig. 3).

### ERβ1 expression using PPG5/10 antibody is not associated with recurrence in TNBC

**Fig. 4:**
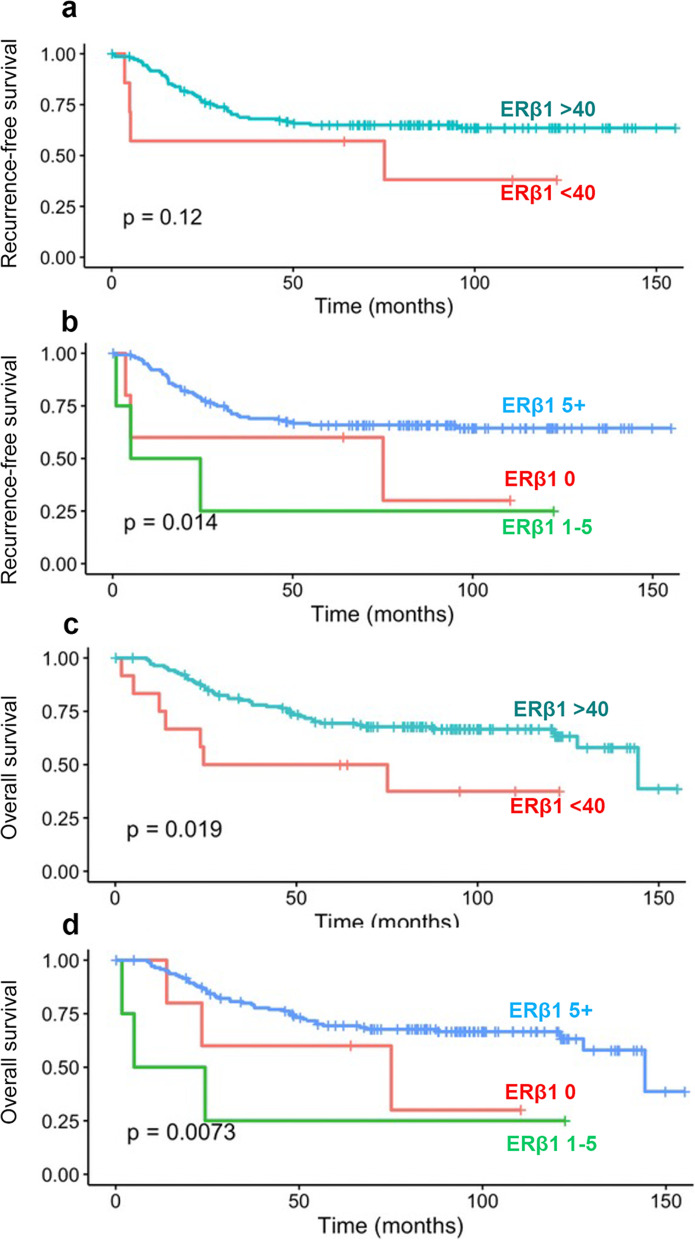
Relationship of ERβ1 expression and recurrence free survival using PPG5-10 antibody. Kaplan Meier curves of recurrence free survival (**a**-**b**) according to the expression of ERβ1 when divided into (**a**) less than (or equal to) 40% or more than 40%. **b** Allred score of 0, 1–5 or > 5. Kaplan Meier curves of overall survival (**c**-**d**) according to the expression of ERβ1 when divided into (**a**) less than (or equal to) 40% or more than 40%. **b** Allred score of 0, 1–5 or > 5

When stained with the PPG5/10 antibody, we found that 88% had ERβ1 high expression (> 40%). Those PPG5/10 ERβ1 high cases were no more likely to experience a recurrence than ERβ1 low cases, *p* = 0.12 (Fig. 4). When we assessed recurrence free survival according to Allred scores, the Allred 1–5 category were more likely to have a recurrence and Allred > 5 had the best prognosis *p* = 0.014. When we assessed overall survival, PPG5/10 ERβ1 high cases (> 40%) were less likely to die compared to ERβ1 low cases, *p* = 0.019. When we assessed overall survival according to Allred scores, the Allred 1–5 category were more likely to die *p* = 0.0073 with Allred > 5 having best prognosis. No associations were found with disease specific survival (data not shown).

Concerns have been raised as to the specificity of the PPG5/10 antibody. Andersson and colleagues showed that PPG5/10 failed immunohistochemical validation as it generated distinct positive staining in ERb negative lines. It also produced strong unspecific bands on western blot. When this was followed up by immunoprecipitation and mass spectroscopy it identified a range of nuclear proteins including the transcriptional activators EWSR1 and YTHDF3. Wu and colleages used doxycycline inducible ERα or ERβ osteosarcoma cell lines [27, 28] and showed in low resolution images that whilst ERb expressing lines were positive, background staining in the control cell lines was also observed. In contrast we have previously shown that the CWK-F12 antibody only showed expression in the ERβ1 overexpressing cell lines [22].

In terms of concordance of the antibodies, The majority (93%) of patients who had high ERβ1expression when stained with the CWK-F12 also showed high expression when stained with the PPG5/10 antibody. For those patients who had low ERβ1 expression with the CWK-F12 antibody, only 8% also showed low expression with the PPG5/10 antibody. A correlation plot shows that there was no correlation between the two antibodies (Supplementary Fig. 4) whether assessed as percententage of expression or Allred score (Spearman’s Rank 0.03 *p* = 0.657 and 0.103, *p* = 0.205 respectively).

## Discussion

In our series of TNBCs nuclear ERβ1was expressed in 72% of cases. This is the first study to assess the expression of ERβ1 in TNBC since the robust validation of the existing CWK-F12 ERβ1 antibody by western blot and RIME analyses [21] as well as our IHC validation [22]. The high proportion of ERβ expression in TNBC in our work is similar to Yan and colleagues who showed that approximately 75% of TNBC were ERβ1 positive, but used the PPG5/10 antibody which has been shown to be non-specific via western blot and RIME analysis. Gruvberger-Saal and colleagues [13] used a cocktail of ERβ antibodies (PP65-10 and 14C8), the former non-specific and the latter specific by western blot, and found a high percentage of ERα negative tumors to be ERβ1 positive (~ 55%). Three other studies have assessed the levels of ERβ1 protein and found 25% of ERα-negative [17], 35.5% of TNBC [16] and 83% of TNBC [14] to be ERβ1 positive. Two of these studies used the PPG5-10 antibody [14, 17]. The polyclonal rabbit antibody used by Guo and colleagues (#BY-02101; Shanghai Yueyan Biological Technology, Co., Ltd., Shanghai, China) has not been assessed for specificity [16]. Regardless, our results shows that a high proportion of TNBC express nuclear ERβ1.

We have demonstrated that the expression of ERβ1 using the CWK-F12 ERß1 antibody was not associated outcome in TNBC. Guo and colleagues found that survival in TNBC cancer patients (*n* = 107) was inferior in those with ERβ expression (χ^2^ = 5.330, *p* = 0.021) [16]. We found a trend for inferior recurrence, but no effect on survival. The discrepancy in the data may be due to the differences in the numbers of patients assessed, and potentially the clinical treatment of the patients. We did not find an effect on overall survival. Our results demonstrate that ERβ1 is not prognostic for recurrence or survival. To assess if downstream ERβ1 specific transcriptional programs were present in *ESR2* + TNBC cancers we performed Pearson correlation analysis between the high *ESR2* expressing group in TNBC and downstream genes known to be upregulated or downregulated following *ESR2* activation [29]. *PROM6*, *FN1* and *SLC16A6* were significantly positively correlated with *ES2R*. *ANKRD35*, *ASB9* and *SELENBP1* were negatively correlated. Whilst this in encouraging and indicates that ERβ1 is driving downstream estrogen actions in TNBC, further work is needed to define what this means functionally for the breast cancer cells.

To allow some comparison of our data with the previously published results using non-validated antibodies, we also stained our cohort with the PPG5-10 antibody and assessed the relationship between ERβ1 expression and prognosis. High ERβ1 (> 40%) expression (compared to low) was not associated with recurrence but an Allred score of > 5 was associated with less recurrence compared to an Allred of 1–5. High ERβ1 (> 40%) expression (compared to low) was associated with better overall survival, as was an Allred of > 5 compared to 1–5. This supports previous reports with this antibody showing that in ERα negative tumors, ERβ1 expression is associated with good prognosis [13, 14]. However, we and others believe this antibody is non-specific and thus do not draw conclusions from its expression and recurrence or survival when tested on the same patient cohort. If further work on this antibody does indicate it is specific, we acknowledge that our work may indicate that full length ERβ, but not splice variants associate with BCa prognosis. At present however, our work cautions against the interpretation of previous data which has used a non-specific ER antibody for staining.

Currently only those women with ERα positive tumors are treated with endocrine therapies such as tamoxifen. TNBC patients lack defined drug targets, and so would benefit greatly from the identification of new targeted therapeutics. There is some indication that ERβ expression may act as a biomarker of tamoxifen sensitivity. In a small cohort of TNBC patients (*n* = 50) who were treated with tamoxifen for two or more years, Honma and colleagues [14] reported that those whose tumors expressed ERβ had significantly longer survival. However, this study used the non-specific PPG5-10 antibody, and did not include a control cohort without tamoxifen treatment. Similarly, Gruvberger-Saal and colleagues found that expression of ERβ was associated with increased survival (distant disease-free and overall survival) in tamoxifen-treated ERα-negative patients but not in the ERα-positive subgroup [13], but again did not evaluate an untreated cohort. In a study assessing ERβ1 expression in tissue microarrays from a randomized, placebo-controlled trial of tamoxifen therapy (NCIC-CTG-MA12), high ERβ1 expression in ERα negative patients was associated with longer recurrence free survival in tamoxifen-treated patients compared to placebo [15]. This study used a polyclonal, GC17/385P, Biogenex ERβ antibody that was shown previously to be specific [30], but was not tested in the recent antibody validation studies. Interestingly, this study demonstrated that in ERα-negative patients, ERβ1-high tumors were associated with a worse outcome (52% 5-year recurrence free survival), which could be improved to a level of survival (77% 5 year survival) very similar to patients with ERβ1-low tumors (75–76%) by tamoxifen treatment.

Our study represents the first essential step towards determining whether ERβ1 expression should be routinely assessed in TNBC in general as a prognostic factor. We find that the often-used PPG5/10 antibody did show associations with recurrence and survival, however these trends were not observed when we used a validated ERβ1 antibody. This highlights the need to re-assess the relationship between ERβ1 expression and tamoxifen sensitivity in TNBC patients that have been treated with Tamoxifen [13–15]. This would then allow the field to determine if ERβ1 has any potential therapeutic target in TNBC as indicated in fulvestrant treated ERβ1 positive TNBC [31].

## Conclusion

In conclusion, using a validated antibody, ERβ1 was not a prognostic indicator in TNBC and indicates that endocrine therapies, or at least ERβ1 specific therapies, may not provide much clinical benefit to this group of patients.

## Supplementary Information

**Additional file 1: Supplementary Fig. 1.** Determining the cut off scores for analysis. Frequency histogram of percentage ERβ1 staining (a) showing distinct clusters and thresholds determined (dotted vertical lines). Frequency histogram of Allred score of ERβ1 staining (determined using a combination of percentage and intensity) (b) showing distinct clusters and thresholds determined (dotted vertical lines).

**Additional file 2: Supplementary Fig. 2.** Log expression of androgen receptor (AR) in gene expression datasets of TNBC according to their annotated TNBC subtypes. Basal-like 1 (BL1), basal-like (BL2), immunomodulatory (IM), mesenchymal (M), mesenchymal stem–like (MSL), luminal androgen receptor (LAR) subtype and unspecified group (UNS). *** *P* < 0.001 HSD test between LAR and other subtypes. Not shown MSL vs IM *p < 0.05 and MSL vs BL1 **p < 0.01.

**Additional file 3: Supplementary Fig. 3**. *a. ESR2* expression (logCPM) by breast cancer subtype. (The centre line of the boxplot indicates the median, the lower and upper hinges correspond to the first and third quartiles. The whiskers extend from the hinges to the largest or smallest value no further than 1.5 * IQR from the hinge. Data beyond the end of the whiskers are individual outliers.) b. Histogram showing *ESR2* expression levels in basal breast cancers (*n* = 173). c. Boxplot showing the *ESR2* expression level classification of basal breast cancers. d. Correlations between *ESR2* and downstream genes in the high *ESR2* group of basal breast cancers.

**Additional file 4: Supplementary Fig. 4.** Jitter XY plot showing the relationship between staining with the CWK-F12 antibody and PPG5-10 antibody as a. the percentage of staining or b. according to Allred score. Spearman’s rank correlation results are included on the graphs.

**Additional file 5: Supplementary Table 1.** Genes known to be specifically up or downregulated in *ESR2* overexpressing MCF7 breast cancer cell lines.

## Data Availability

All data generated or analysed during this study are included in this published article and its supplementary information files.

## Abbreviations

BCa: Breast Cancer
ER: Estrogen receptor
ERβ: Estrogen receptor beta
LN: Lymph node
PR: Progesterone Receptor
RIME: Rapid immunoprecipitation mass spectrometry of endogenous protein
SERM: Selective ER modulator
TMA: Tissue microarray
TNBC: Triple negative breast cancer
