# Supplementary figures and images for: Estrogen receptor beta expression in triple negative breast cancers is not associated with recurrence or survival
Source: BMC Cancer. 2023 May 19;23:459. doi: 10.1186/s12885-023-10795-5 (PMC10197848; doi:10.1186/s12885-023-10795-5)

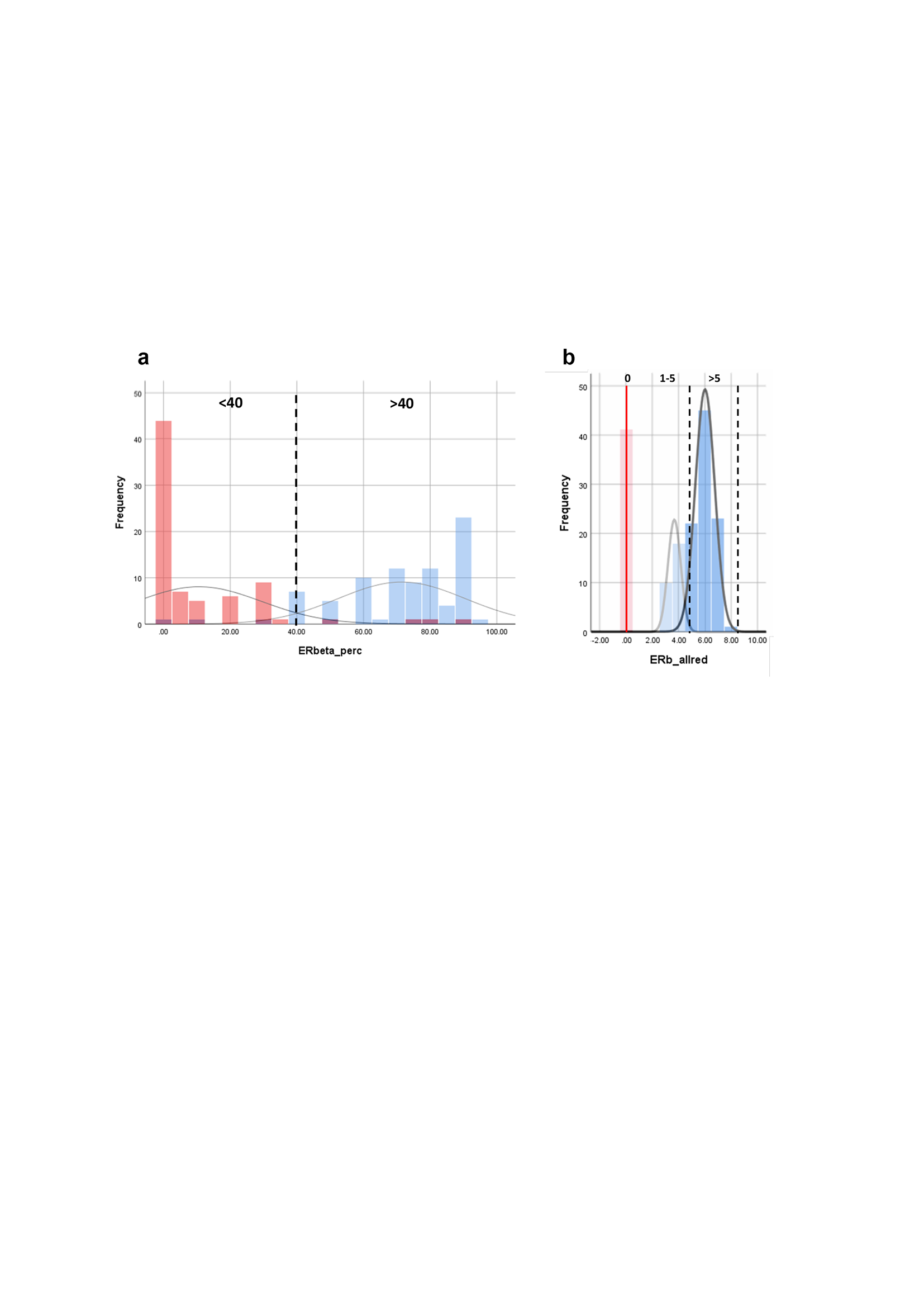

Supplement: Supplementary file 1 — Additional file 1: Supplementary Fig. 1. Determining the cut off scores for analysis. Frequency histogram of percentage ERβ1 staining (a) showing distinct clusters and thresholds determined (dotted vertical lines). Frequency histogram of Allred score of ERβ1 staining (determined using a combination of percentage and intensity) (b) showing distinct clusters and thresholds determined (dotted vertical lines). [file 12885_2023_10795_MOESM1_ESM.tif]

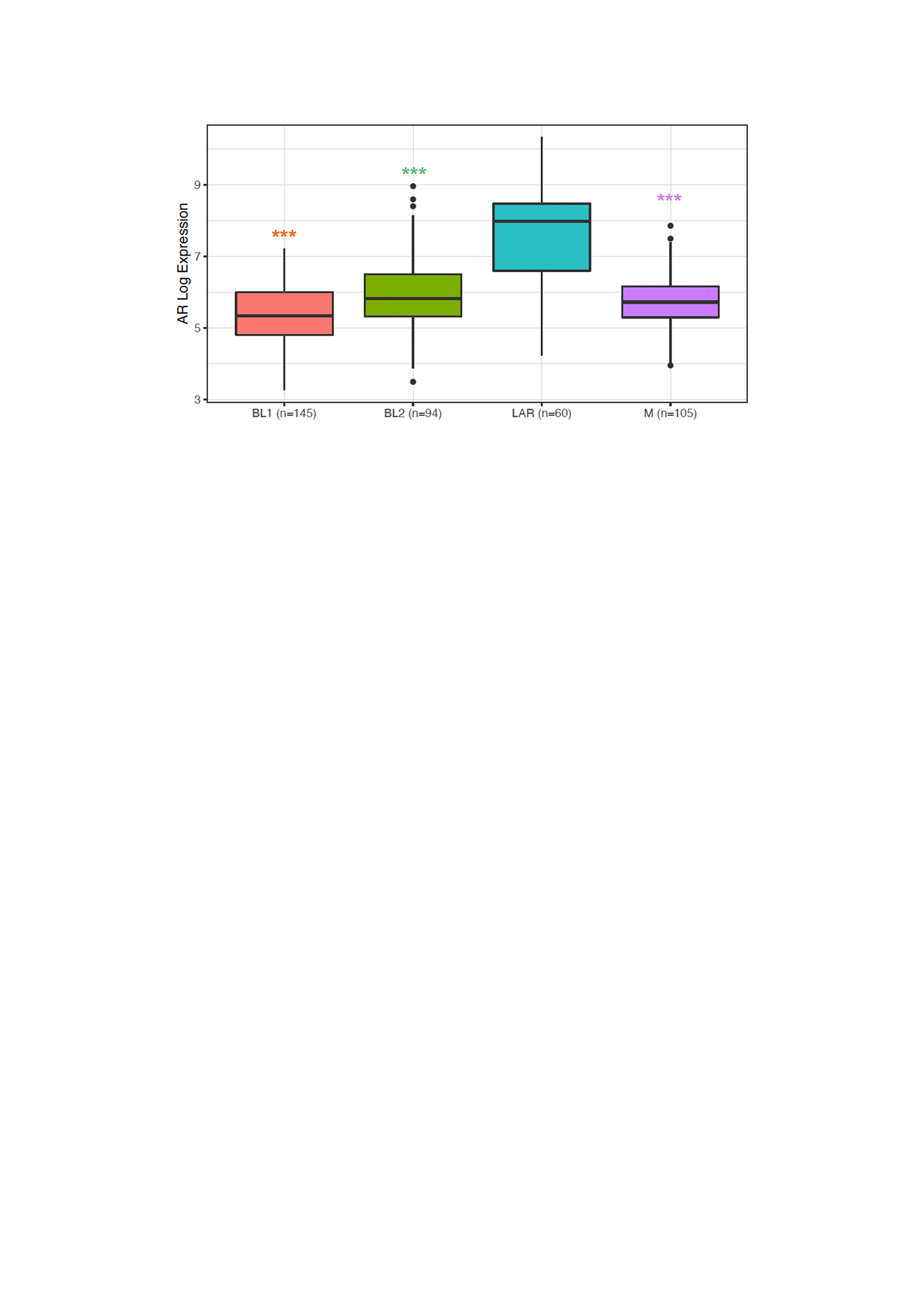

Supplement: Supplementary file 2 — Additional file 2: Supplementary Fig. 2. Log expression of androgen receptor (AR) in gene expression datasets of TNBC according to their annotated TNBC subtypes. Basal-like 1 (BL1), basal-like (BL2), immunomodulatory (IM), mesenchymal (M), mesenchymal stem–like (MSL), luminal androgen receptor (LAR) subtype and unspecified group (UNS). *** P < 0.001 HSD test between LAR and other subtypes. Not shown MSL vs IM *p < 0.05 and MSL vs BL1 **p < 0.01. [file 12885_2023_10795_MOESM2_ESM.tif]

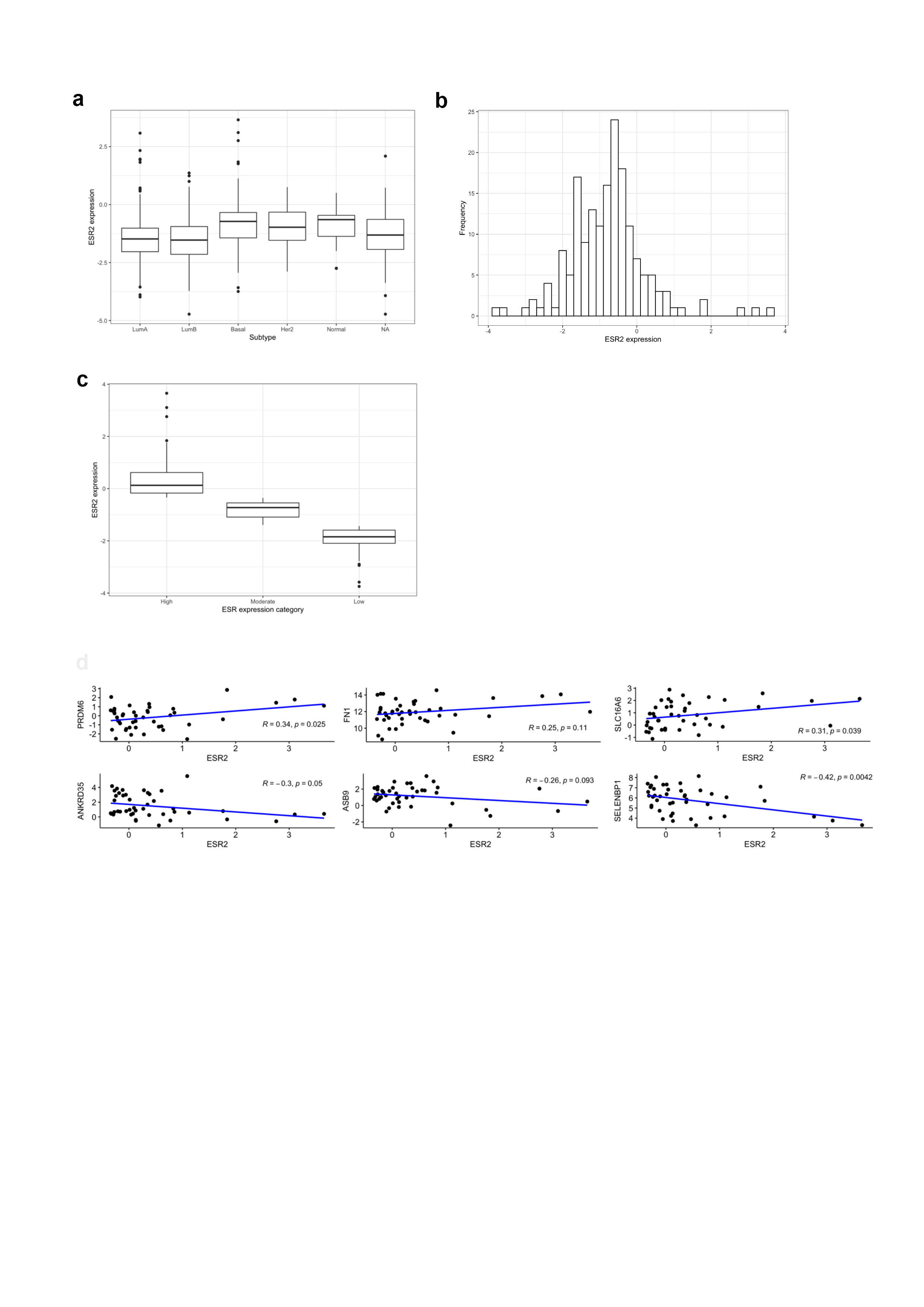

Supplement: Supplementary file 3 — Additional file 3: Supplementary Fig. 3. a. ESR2 expression (logCPM) by breast cancer subtype. (The centre line of the boxplot indicates the median, the lower and upper hinges correspond to the first and third quartiles. The whiskers extend from the hinges to the largest or smallest value no further than 1.5 * IQR from the hinge. Data beyond the end of the whiskers are individual outliers.) b. Histogram showing ESR2 expression levels in basal breast cancers (n = 173). c. Boxplot showing the ESR2 expression level classification of basal breast cancers. d. Correlations between ESR2 and downstream genes in the high ESR2 group of basal breast cancers. [file 12885_2023_10795_MOESM3_ESM.tif]

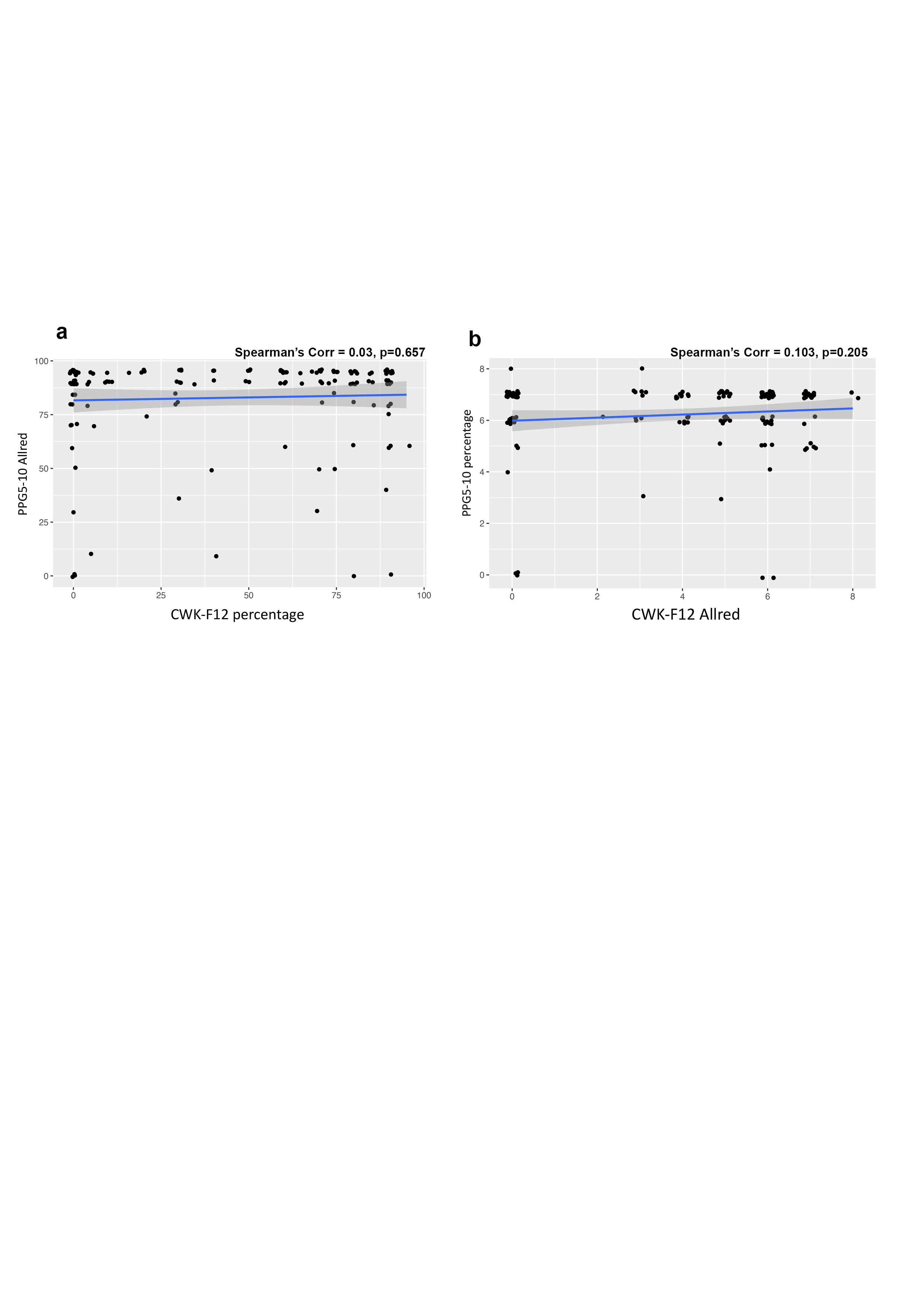

Supplement: Supplementary file 4 — Additional file 4: Supplementary Fig. 4. Jitter XY plot showing the relationship between staining with the CWK-F12 antibody and PPG5-10 antibody as a. the percentage of staining or b. according to Allred score. Spearman’s rank correlation results are included on the graphs. [file 12885_2023_10795_MOESM4_ESM.tif]
